# Admission Hypothermia and Neonatal Mortality in the Ethiopian Neonatal Network

**DOI:** 10.1177/30502225251364989

**Published:** 2025-08-22

**Authors:** Kullehe Haddis Yeshanew, Asrat Demtse, Mahlet Abayneh, Danielle E. Y. Ehret, Firehiwot Markos Mekuria, Erika M. Edwards

**Affiliations:** 1 Addis Ababa University, Ethiopia; 2 St Paul’s Hospital Millenium Medical College, Addis Ababa, Ethiopia; 3 Vermont Oxford Network, Burlington, VT, USA; 4 University of Vermont, Burlington, VT, USA

**Keywords:** infant, newborn, hypothermia, Ethiopia, neonatology

## Abstract

**Background:**

Admission hypothermia remains a problem in low-income countries.

**Methods:**

This cross-sectional study evaluated the association between admission hypothermia (<36.5°C) and neonatal mortality for newborns discharged from neonatal units at 20 Ethiopian hospitals in 2021.

**Results:**

Among 12 363 newborns, 51.6% were admitted with hypothermia. Predictors for hypothermia and mortality included multiple birth, low birth weight, lower gestational age, and congenital anomalies. In a multivariable analysis, risk for mortality increased for infants admitted at <33.9°C [aRR 2.39 (95% CI: 1.66, 3.45)], 34.0°C to 34.9°C [aRR 2.45 (95% CI: 1.88, 3.20)], 35.0°C to 35.9°C [aRR 2.00 (95% CI: 1.46, 2.74)], and 36.0°C to 36.4°C [aRR 1.59 (95% CI: 1.24, 2.04)], compared to infants admitted at 36.5°C to 37.5°C.

**Conclusions:**

Admission hypothermia was associated with a twofold increased risk of death. To address hypothermia, basic essential newborn care, proper warm intra-facility transportation, improved infrastructure and equipment, and skin-to-skin care should be prioritized.

## Introduction

Globally, in 2022, 2.3 million deaths occurred in newborns less than 28 days from birth. Sub-Saharan Africa had the highest neonatal mortality rate, of 27 per 1000 live births. Most of the newborns (75%) died in the first 1 week of life and 1 million of those died in the first 24 hours of life.^1^

Maintaining a normal body temperature is an important aspect of newborn survival.^2^ The World Health Organization (WHO) defined hypothermia as a core body temperature less than 36.5 degrees Celsius (°C) and classified hypothermia as mild (36°C-36.4°C), moderate (32°C-35.9°C) and severe (<32°C).^3^ Although it is known that hypothermia increases the risk of both mortality and morbidity, a direct causal relationship has not been identified.^2,4-6^

Hypothermia remains a global problem despite the different mechanisms proposed to prevent it.^1,2,4^ According to a systematic review, the global prevalence of hypothermia in hospitals ranged between 32% and 85% and that of home delivery was between 11% and 92%, even in tropical environments.^7^ In another study, the body temperature of newborns less than 36°C in Nepal was found to be 85% in newborns delivered in a hospital within the first 2 hours of life.^8^ Similarly, in some parts of Sub-Saharan Africa, an incidence of 65% to 85% has been documented.^5^ In a study done by Demtse et al in 2020, the prevalence of hypothermia in preterm infants in 5 specialized hospitals of Ethiopia was found to be 79.5% and as the degree of hypothermia worsened, the severity of morbidity and mortality increased.^6^ In very low birth weight infants, for every one-degree Celsius decrease in body temperature less than 36°C, mortality rates have been shown to increase by 28%.^4,6^

Several studies have examined hypothermia in Ethiopia; however, most are single center observations or focus on a localized geographic area.^6,9-15^ Admission hypothermia has not previously been examined on a large scale, especially in community hospitals representing all regions of the country. We used data from the Ethiopian Neonatal Network (ENN) to assess the prevalence of hypothermia on admission to the neonatal unit and its association with mortality to provide more generalized, country-wide information.

## Methodology

**Figure 1.**
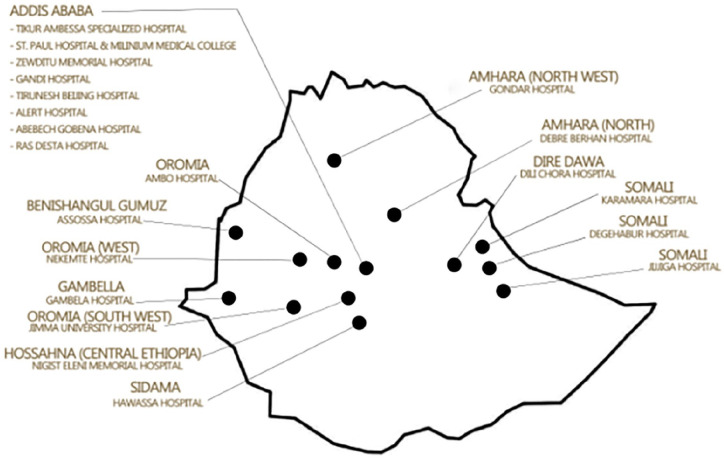
Map of Ethiopian Neonatal Network hospitals included in the study.

Twenty hospitals in Ethiopia were involved in the study. The ENN was established in 2018 in collaboration with Vermont Oxford Network (VON), Ethiopian Pediatric Society, and the Ethiopian Federal Ministry of Health. VON is a voluntary worldwide community of practice dedicated to improving the quality, safety, and value of newborn care through a coordinated program of data-driven quality improvement, education, and research.^16^ The goal of the ENN is to establish data driven quality improvement among member hospitals. The ENN constitutes 6 hospitals from Addis Ababa, 3 hospitals from Oromia, 2 hospitals from Amhara region, 3 from Somali region, and 1 hospital each from Tigray, Benishangul Gumuz, Diredawa, Gambella, Central Ethiopia, and Sidama regions (Figure 1). There are 15 general/community hospitals and 5 specialized hospitals with 16 level II and 4 level III neonatal intensive care units (NICUs).

This study is a secondary analysis of prospectively collected data from the ENN database for all infants discharged from a neonatal unit from January 1, 2021, to December 31, 2021. Data were entered by the local staff using standardized definitions.^17^ Anatomical location and method of temperature measurement were not recorded. All infants whose temperature was recorded within the first hour of birth and whose outcomes were known were included in the study.

Associations were tested for mode of delivery (vaginal/cesarean section), birth weight, place of delivery (inborn/outborn), whether the newborn was term or preterm (<37 weeks’ gestation), average outside environmental temperature ≥25°C or <25°C),^18^ season of the year the newborn was born, the day the patient was born (weekday/weekend), and the hospital’s level of neonatal care. Season was defined as wet (June, July, August) and dry (September, October, November, December, January, February, March, April, May). The levels of the hospitals and NICUs were made based on the draft Ethiopian Ministry of Health classification system (unpublished). The outside environmental temperatures were classified based on the average annual environmental temperature for the hospital’s geographic location. Common discharge diagnoses (perinatal asphyxia, respiratory distress, late onset sepsis, or meconium aspiration syndrome) were selected to assess effect modification between admission hypothermia and mortality.

Risk ratios (RR) and 95% confidence intervals (95% CI) for mortality were estimated in univariable and multivariable analyses using generalized estimating equation logistic regression models with a log link to control for clustering of infants within hospitals. Only variables with statistically significant associations in univariable analyses were entered into the multivariable analysis. We assessed whether admission temperature was an effect modifier between common discharge diagnoses and mortality; a *P*-value of <.05 indicated an interaction. All analyses were done using SAS 9.4.

### Ethical Approval and Informed Consent

Ethical clearance for this analysis was obtained from the Addis Ababa University School of Medicine, College of Health Sciences Pediatrics and Child Health Department’s Research and Publications Committee (DRPC/007/16). All 20 sites received approval from their local IRBs to participate in the ENN. The University of Vermont institutional review board determined that the ENN was exempt with a waiver of informed consent (CHRMS 18-0009). The authors used the Projects Requiring Review tool provided by the University of Vermont institutional review board to determine that this analysis was not human subjects research.

## Results

Among the 12 363 infants included in the study, 28.3% were born preterm (<37 weeks), 35.3% were low birth weight (≤2500 g), and 7% were very low birth weight (≤1500 g; Table 1). More than half of the infants (57.3%) were male and 7.9% of the deliveries were multiple births. Maternal age was 26 to 35 years for 50.1% of the cases.

**Table 1.** Maternal and Infant Characteristics Among 12 363 Infants.

Characteristics	Numerator	%
Maternal age		
<18 years	65	0.5
18-25 years	5411	43.8
26-35 years	6194	50.1
>35 years	689	5.6
Gestational age		
<30 weeks	431	3.5
31-33 weeks	983	8.0
34-36 weeks	2080	16.8
37-39 weeks	6204	50.2
≥40 weeks	2665	21.6
Birth weight		
<1500 g	870	7.0
1500-1999 g	1712	13.9
2000-2499 g	1785	14.4
2500-2999 g	2768	22.4
3000-3499 g	3142	25.4
≥3500 g	2080	16.8
Male sex	7081	57.3
Multiple birth	980	7.9
Congenital anomaly	861	7.0

**Figure 2.**
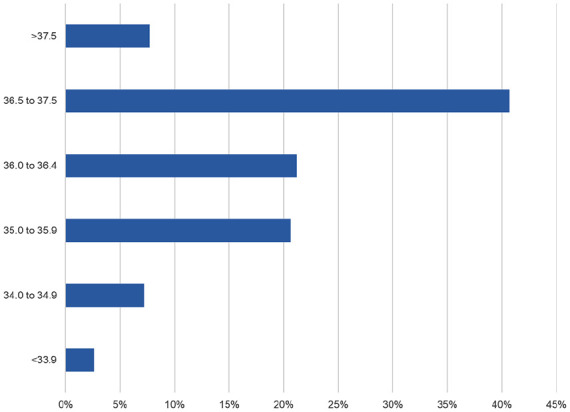
Distribution of admission temperature.

The most frequent admission body temperature was normothermia, defined as 36.5°C to 37.5°C (40.7%), followed by 36.0°C to 36.4°C (21.2%), 35.0°C to 35.9°C (20.6%), 34.0°C to 34.9°C (7.2%) and <33.9°C (2.6%) (Figure 2). Hyperthermia (temperature >37.5°C) was recorded in 7.7% of the infants.

Predictors of hypothermia included being inborn, being part of a multiple birth, born preterm, or born low birth weight (Table 2). Additionally, infants born at level III units were more likely to be hypothermic. The hypothermia rates were similar across the dry and wet seasons, by type of delivery, presence of congenital anomaly, the hospital’s outside environmental temperature, or whether the infant was born during the week or on the weekend.

**Table 2.** Risk Factors for Hypothermia by Admission Temperature.

	Temperature 36.5-37.5 (N = 5029)		Temperature < 36.5 (N = 6385)	
Covariates	Numerator	%	Numerator	%
Mode of delivery				
Vaginal	3574	71.1	4399	69.0
Cesarean	1454	28.9	1977	31.0
Place of delivery				
Outborn	1195	23.8	1182	18.5
Inborn	3829	76.2	5200	81.5
Gestation (weeks)				
<30	73	1.5	346	5.4
31-33	243	4.8	726	11.4
34-36	601	12	1425	22.3
≥37	4112	81.8	3888	60.9
Birth weight (g)				
<1500	179	3.6	682	10.7
1500-1999	498	9.9	1171	18.4
2000-2499	596	11.9	1089	17.1
≥2500	3756	74.7	3438	53.9
Type of birth				
Multiple	277	5.5	685	10.7
Singleton	4748	94.5	5695	89.3
Congenital anomaly				
Yes	370	7.4	389	6.1
No	4651	92.6	5984	93.9
Outside environmental temperature				
<25	4337	86.2	4959	77.7
≥25	692	13.8	1426	22.3
Level of unit				
III	1376	27.4	2584	40.5
II	3653	72.6	3801	59.5
Month of birth				
Wet season	1256	25	1430	22.4
Dry season	3773	75	4955	77.6
Date of birth				
Weekday	3696	73.5	4683	73.3
Weekend	1333	26.5	1702	26.7

Independent risk factors for mortality are reported in Table 3. The risk of death increased more than 5 times [RR 5.65 (95% CI: 4.31, 7.41)] when the admission temperature was less than 33.9°C, more than 4 times [RR 4.25 (95% CI: 3.30, 5.49)] when the temperature was 34°C to 34.9°C, close to 3 fold when the temperature was 35°C to 35.9°C [RR 2.83 (95% CI: 2.16, 3.71)] and 2 times when the temperature was 36°C to 36.4°C [RR 1.83 (95% CI: 1.43, 2.33)] as compared to those with normal temperature. There was no increased risk associated with hyperthermia. Other predictors of mortality included lower gestational age, lower birth weight, multiple birth, and presence of a congenital anomaly. Inborn infants had reduced risk of mortality. NICU level, outside environmental temperature, season of the year or whether the newborn was born on a weekday or weekend were not significantly associated with mortality.

**Table 3.** Independent Risk Factors for Mortality.

	Survived (N = 11 091)		Did not survive (N = 1272)		RR (95% CI)
Covariates	Numerator	%	Numerator	%	
Mode of delivery					
Cesarean	3328	30	335	26.3	0.84 (0.71, 1.01)
Vaginal	7753	70	937	73.7	Reference
Place of delivery					
Outborn	2360	21.3	373	29.3	1.46 (1.16, 1.84)
Inborn	8721	78.7	899	70.7	Reference
Gestation					
<30 week	183	1.6	248	19.5	9.87 (7.99, 12.20)
31-33 week	702	6.3	281	22.1	4.90 (3.77, 6.37)
34-36 week	1854	16.7	226	17.8	1.86 (1.45, 2.39)
≥ 37 week	8352	75.3	517	40.6	Reference
Birth weight					
<1500 g	443	4	427	33.6	9.57 (7.92, 11.55)
1500-1999 g	1443	13	269	21.2	3.06 (2.37, 3.95)
2000-2499 g	1620	14.6	165	13	1.80 (1.46, 2.23)
≥2500 g	7580	68.4	410	32.3	Reference
Type of birth					
Multiple	808	7.3	172	13.6	1.82 (1.45, 2.30)
Singleton	10 279	92.7	1095	86.4	Reference
Congenital anomaly					
Yes	674	6.1	187	14.7	2.30 (1.60, 3.30)
No	10 398	93.9	1084	85.3	Reference
Environmental temperature					
<25	9014	81.3	1027	80.7	0.97 (0.59, 1.61)
>25	2077	18.7	245	19.3	Reference
Level of unit					
3	3758	33.9	457	35.9	1.08 (0.72, 1.64)
2	7333	66.1	815	64.1	Reference
Month of birth					
Wet season	2585	23.3	280	22	0.94 (0.81, 1.09)
Dry season	8506	76.7	992	78	Reference
Date of birth					
Weekend	2976	26.8	333	26.2	1.03 (0.91, 1.17)
Weekday	8115	73.2	939	73.8	Reference
Admission temperature					
<33.9	225	2	97	7.6	5.65 (4.31, 7.41)
34-34.9	689	6.2	202	15.9	4.25 (3.30, 5.49)
35-35.9	2167	19.5	385	30.3	2.83 (2.16, 3.71)
36-36.4	2365	21.3	255	2	1.83 (1.43, 2.33)
36.5-37.5	4761	42.9	268	21.1	Reference
>37.5	884	8	65	5.1	1.29 (0.91, 1.81)

In the multivariable analysis controlling for place of birth, birth weight, mode of delivery, gestational age and type of birth, compared to infants admitted with normal temperature, the risk of death was at least 2 times higher for infants with moderate or severe hypothermia on admission: <33.9°C [aRR 2.39 (95% CI: 1.66, 3.45)], 34.0°C to 34.9°C [aRR 2.45 (95% CI: 1.88, 3.20)], 35.0°C to 35.9°C [aRR 2.00 (95% CI: 1.46, 2.73)]. Additionally, the risk of death was 1.6 times higher for mild hypothermia, 36.0°C to 36.4°C [aRR 1.59 (95% CI: 1.24, 2.04)] (Table 4). Birth weight and presence of a congenital anomaly also increased risk of mortality, while inborn status decreased risk (Table 4).

**Table 4.** Multivariable Analysis of Risk Factors for Mortality.

Covariates	RR (95% CI)
Admission temperature	
<33.9	2.39 (1.66, 3.45)
34-34.9	2.45 (1.88, 3.20)
35-35.9	2.00 (1.46, 2.73)
36-36.4	1.59 (1.24, 2.04)
36.5-37.5	Reference
>37.5	1.40 (0.99, 1.99)
Place of delivery	
Outborn	1.41 (1.16, 1.71)
Inborn	Reference
Birth weight	
<1500 g	8.33 (6.98, 9.94)
1500-1999 g	2.88 (2.30, 3.61)
2000-2499 g	1.66 (1.35, 2.04)
≥2500 g	Reference
Type of birth	
Multiple	0.89 (0.72, 1.10)
Singleton	Reference
Congenital anomaly	
Yes	2.67 (1.99, 3.58)
No	Reference

Admission temperature was not an effect modifier in the associations between perinatal asphyxia, respiratory distress, or meconium aspiration syndrome and mortality (Table 5). However, admission temperature was an effect modifier in the association between any sepsis and mortality. Infants who were hypothermic on admission and developed sepsis had higher risk of mortality [aRR 1.94 (95% CI: 1.42, 2.67)] than infants who were normothermic [aRR 1.47 (95% CI: 0.95, 2.27)].

**Table 5.** Effect Modification of Admission Temperature on Risk of Mortality for Specific Discharge Diagnoses.

	Temperature 36.5-37.5 (N = 5029)					Temperature < 36.5 (N = 6385)				
	Survived (N = 4761)		Did not survive (N = 268)		aRR (95% CI)	Survived (N = 5446)		Did not survive (N = 939)		aRR (95% CI)
Morbidities	Numerator	%	Numerator	%		Numerator	%	Numerator	%	
Perinatal asphyxia										
Yes	176	3.7	7	2.6	0.71 (0.25, 2.05)	193	3.5	28	3	0.86 (0.58, 1.28)
No	4579	96.3	260	97.4	Reference	5247	96.5	910	97	Reference
Respiratory distress										
Yes	1909	40.1	186	69.4	3.18 (2.14, 4.72)	2839	52.1	732	78	2.79 (2.05, 3.78)
No	2851	59.9	82	30.6	Reference	2606	47.9	207	22	Reference
Sepsis										
Yes	3452	72.6	214	79.9	1.47 (0.95, 2.27)	4088	75.1	811	86.6	1.94 (1.42, 2.67)
No	1306	27.4	54	20.1	Reference	1354	24.9	126	13.4	Reference
Meconium aspiration syndrome										
Yes	750	15.8	49	18.3	1.18 (0.79, 1.77)	965	17.7	159	17	0.95 (0.65, 1.41)
No	4008	84.2	219	81.7	Reference	4473	82.3	778	83	Reference

## Discussion

This study found that the proportion of 12 363 infants admitted to neonatal units in Ethiopia with hypothermia was 51.6%, and neonatal mortality was increased at least twice for admission temperatures less than 36.5°C. A number of studies have previously been done on hypothermia in different regions of Ethiopia, with limited sample sizes.^6,9-15,19,20^ This study included 20 hospitals distributed all over the country with different geographic and cultural structures. The large sample size used with representation from every region of the country gives more generalizable information about the situation of admission hypothermia in Ethiopia.

The rate of hypothermia in our study is similar to that published in a systematic review and meta-analysis by Beletew et al in 2020 where Ethiopia had a prevalence of 55%^21^ and a 2021 Ugandan study where the prevalence of hypothermia was 51%.^22^ Our result also lies in the range of the percentages mentioned in a systematic review by Lunze et al where the prevalence of hypothermia in low- and middle-income countries was determined to range between 32% and 85%.^7^ We confirmed that being part of a multiple birth, low birth weight, and having a lower gestational age at birth were associated with lower admission temperatures which is consistent with a number of studies in Ethiopia and other sub-Saharan African countries.^4,6,9-15,19-25^

Inborn infants were more likely to be hypothermic than outborn infants in our study. This result is different from a study done in Nigeria^23^ where the rate of hypothermia was higher in the outborn population (64.4% vs 58.3%) and in a previous study in Ethiopia by Demtse et al where outborns had a higher prevalence of hypothermia even though it was not statistically significant.^6^ However, this finding of higher prevalence of hypothermia in inborn infants was also seen in a study done in Ghana.^24^ The higher prevalence of hypothermia in inborn infants might be due to the poor follow up of the warm chain, lack of knowledge about the dangers of hypothermia, lack of warm intra-facility transport and sub-optimal essential newborn care practices done in the hospitals, and lack of adequate equipment for maintaining normothermia.^2,3,5,25,26^ Additional risk factors for hypothermia may include a high ratio of patients to midwives, and more high-risk and low birth weight infants being born in hospitals instead of in health clinics or at home. The hypothermia rate in level III NICUs was also higher although there was no significant association between NICU level and mortality when comparing level II and level III neonatal units. The level III NICUs are referral centers that are more likely to provide care for sick, preterm, and low birth weight babies who also have an increased risk of becoming hypothermic.^4,6^

In our 20 hospitals, there was no increased risk of being hypothermic when born in either different seasons of the year or different environmental temperatures. A systematic review done by Kumar et al identified seasonal differences in ambient temperatures led to variation in hypothermia incidence.^8^ A study in Ghana found that the rate of hypothermia was lower during dry seasons as compared to wet seasons.^25^ However, our finding is similar to other studies which state that hypothermia remains a problem in tropical climates.^5,7,27,28^ In addition, our study did not show any difference in hypothermia rates between vaginal or cesarean section deliveries, unlike research done in Indonesia where newborns born vaginally had a 1.5 times lower risk of hypothermia as compared to cesarean section deliveries^27^ and in Ghana where the newborns born vaginally had 40% less risk of developing hypothermia.^25^ Our result is similar to a study in high- and middle-income countries where the hypothermia rate was not affected by the mode of delivery.^4^ A similar hypothermic rate might be due to low delivery room temperatures and cold operation theaters as well as poor practice of skin to skin in both of the delivery situations which will affect thermoregulation in the baby.^4,5,28,29^

When we evaluated admission hypothermia as an independent risk factor, we found that as the degree of admission hypothermia decreased by 1°C below 36.5°C, the outcome of mortality increased by 2 to 5 times compared to those with a normal temperature. This result is consistent with a study done in Brazil where admission temperature in a range of 32.0°C to 35.9°C was associated with 3 times the odds of neonatal death.^30^ We chose to evaluate each degree separately because the WHO classification of moderate hypothermia, 32.0°C to35.9°C, is broad.^3^ We wanted to evaluate the association of each degree difference on mortality.

In multivariable analysis, with the different degrees of hypothermia and controlling for known confounders, the risk of death was consistently increased by 2 times, similar to other studies. Our result is lower than the studies done in Guinea-Bissau and Iran which found that the risk of death increased by 5-fold and 3-fold for neonatal hypothermia respectively.^31,32^ The difference might be due to the classification of the hypothermia in the Guinea study at <34.5°C, lower than our cut-off of ≤36.5°C.^31^ The classification of moderate hypothermia by WHO is wide. Sub-classifying the moderate hypothermia group might help in distinguishing severity as suggested by previous authors.^6,31^

In our study, admission temperature was not an effect modifier in the associations between mortality and perinatal asphyxia, respiratory distress syndrome, or meconium aspiration syndrome. However, admission temperature was an effect modifier in the association between sepsis and death. Infants who were hypothermic at admission and developed sepsis had a significantly increased risk of mortality; infants who were normothermic at admission and developed sepsis did not. Hypothermia influences the ability of the body to fight infections because it can compromise the activity of white blood cells. Hypothermia also reduces the body’s ability to produce and distribute surfactant and resultant pulmonary vasoconstriction leading to increased susceptibility to infections and respiratory distress together with other multi system effects.^2,4,5,33^ The risk of death among infants with respiratory distress was significantly increased regardless of whether the infant was normothermic or hypothermic.

This study includes over 12 000 infants at 20 hospitals with varying levels of neonatal care. The data collectors used standardized definitions, and during this study period, data collection was facilitated by local stipends for data entry, increasing the opportunities for complete and accurate data collection for use in quality improvement activities. Although this is one of the most comprehensive reports on admission hypothermia, limitations exist. Our study reports on patients who were admitted to a neonatal unit in one of the 20 ENN hospitals, were recorded in the database, and had their temperature measured within 1 hour of birth. This study did not include all hospitals in Ethiopia, and did not include patients born at health centers or home births that were not transported to a participating ENN hospital.

## Conclusions

We found that admission hypothermia is prevalent in Ethiopia and mortality even after adjustment for confounders is increased at least 2 times in newborns who had admission hypothermia. Inborn babies and infants cared for at level III NICUs had higher rates of hypothermia compared to outborn and those cared for at level II units. To achieve reductions in newborn mortality by 2030, quality improvement activities should be conducted in all facilities to improve admission hypothermia.^34,35^ Basic essential newborn care, proper warm intra-facility transportation, and kangaroo mother care should be strengthened in addition to improving infrastructure and basic equipment.
